# Promoting Peer Interaction and Acceptance Among Students with Special Needs Through an Experiential Learning Program

**DOI:** 10.3390/children12050543

**Published:** 2025-04-24

**Authors:** Hsiu-Ming Lin, Szu-Yin Chu, Wen-Hsuan Chang, I-Hsuan Lo, Hsin-Ting Peng

**Affiliations:** 1 Department of Special Education, National Tsing Hua University, Hsinchu 300044, Taiwan; emely21481@tmail.hc.edu.tw (H.-M.L.); lihsuan20058@gapp.nthu.edu.tw (I.-H.L.); saskia12tw@gapp.nthu.edu.tw (H.-T.P.); 2 Department of Educational Psychology, Texas A& M University, College Station, TX 77840, USA; wenhsuan.chang@tamu.edu

**Keywords:** experiential learning, peer interaction, acceptance, students with special needs

## Abstract

Background/Objectives: Inclusive education emphasizes positive interactions between students with and without special needs to foster mutual understanding and acceptance. This study explored the effect of an experiential learning program (ExL Prog) on interaction and acceptance between students with and without special needs. Based on Kolb’s ExL theory, this study explores how the ExL Prog fosters experiential learning, reflection, and mutual growth through activities that enhance interpersonal skills, promote empathy, and create an inclusive classroom environment where students with and without special needs deepen their understanding and acceptance of one another. Methods: The study used a mixed-methods approach with 2 students with special needs and 16 students without special needs from the same sixth-grade class. Researchers divided participants into two groups: one intervention group that underwent the 8-activity ExL Prog and one comparison group that participated in regular activities. Data collection methods included questionnaires, interviews, and feedback forms, which enabled qualitative and quantitative analysis. Results: The results indicated the implementation of the ExL Prog facilitated increased opportunities for positive interactions between students with and without special needs. Regarding attitudes of acceptance toward students with special needs, students without special needs in the EXL group demonstrated lower scores than those in the control group in the cognitive dimension, higher scores in the affective dimension, and no significant difference in the behavioral dimension. The program fostered mutual listening, respect, and understanding, particularly among the intervention group, which promoted empathy and supportive relationships among participants. Conclusions: The students in the ExL Program developed a greater understanding of others and reflected on improving their relationship-building approaches. This transformation fostered increased openness, goodwill, and mutual reciprocity, promoting growth in interpersonal relationships and communication skills.

## 1. Introduction

Interpersonal interaction is crucial for developing social skills, communication abilities, understanding of others, peer emotional connections, and acceptance of peers with different traits among school-age children [1,2,3]. The current trend in inclusive education policies is the integration of students with special needs into general education classrooms alongside students without special needs. This practice aims to achieve educational equity and foster broad understanding and interaction between students with special needs and their peers. They promote shared learning, raise awareness of the difficulties of others, enhance understanding of different perspectives, and increase empathy, all contributing to a positive cycle of interpersonal interaction for both parties [4,5,6]. However, integrating students with special needs into general education classrooms often presents challenges in peer interaction and acceptance [6,7]. Even when provided with opportunities for peer relationships, students with special needs may encounter obstacles such as negative experiences and biases related to their disability categories or severity of their disability [8,9]. Therefore, during this integrating process, addressing the negative experiences and biases and identifying positive interpersonal relationships that mutually benefit students with special needs and students without special needs is essential.

Drawing on empirical research and theories of interpersonal development, scholars advocate for experiential learning (ExL) as a potent approach for mitigating negative peer interactions and promoting acceptance [10]. This study is based on Kolb’s [11] ExL theory, where learners engage in experiential activities to gain experiences, then reflect on their feelings and actions from those experiences to generate new ideas. ExL refers to generating reflections through experiences while integrating students’ prior knowledge, enabling them to become active participants in life [12]. ExL promotes direct engagement in activities, empowering individuals to cultivate crucial interpersonal skills through hands-on experiences. Through the ExL program (ExL Prog), individuals reflect on their interactions with others, prompting synthesis and reinterpretation of these experiences. This process enables them to develop interpersonal sensitivity, nurture empathy, and forge deeper, more meaningful connections with others [11,13,14,15]. In the ExL Prog, students with special needs and their peers jointly engage in activities [16]. It promotes mutual growth and creates an inclusive classroom environment in which all students feel accepted and valued. Consequently, it nurtures richer interpersonal connections, encourages positive interactions, and deepens understanding and acceptance between students without special needs and those with special needs.

### 1.1. Peer Interaction and Acceptance

Experiences of recognition and acceptance by peers are vital for both students with and without special needs. A harmonious social integration network fosters a positive peer atmosphere, broadens individual interpersonal resources, and supports the development of mature interpersonal skills and a strong social self-concept [17,18]. Positive peer interaction involves establishing confident and constructive relationships that enrich interpersonal experiences and promote harmony [3]. Inclusive education aims to create a mutually supportive community between students without special needs and those with special needs [5], which benefits the psychological well-being and social development of both groups, breaks down barriers, and promotes mutual growth.

Peer acceptance is a type of attitude that varies in the degree to which someone accepts individual differences among their peers, including different behaviors, traits, and backgrounds. In interpersonal interactions, everyone holds certain attitudes and standards towards other members of their peer group. Students with special needs, characterized by unique behavioral expressions or cognitive–emotional traits [7,19,20] may face challenges in forming stable friendships [21] and may experience peer rejection or social isolation in inclusive education settings [22,23,24].

In brief, positive interactions and acceptance, exemplified by experiential and friendly engagement, are crucial for nurturing mutual peer relationships between students without special needs and those with special needs. Favorable peer relationships aid in resolving interpersonal problems along with empathy and sharing values. In inclusive education settings, interpersonal issues often arise from a lack of understanding. Therefore, ExL Progs are designed to help individuals understand the significance of teamwork and enhance their comprehension of others. These activities also encourage individuals to embrace everyone’s uniqueness with an open mind [25,26,27]. This, in turn, enables them to engage actively and effectively in various social and interpersonal settings, contributing positively to their connections and affairs.

### 1.2. Theoretical Foundations of ExL Cycle

**Figure 1:**
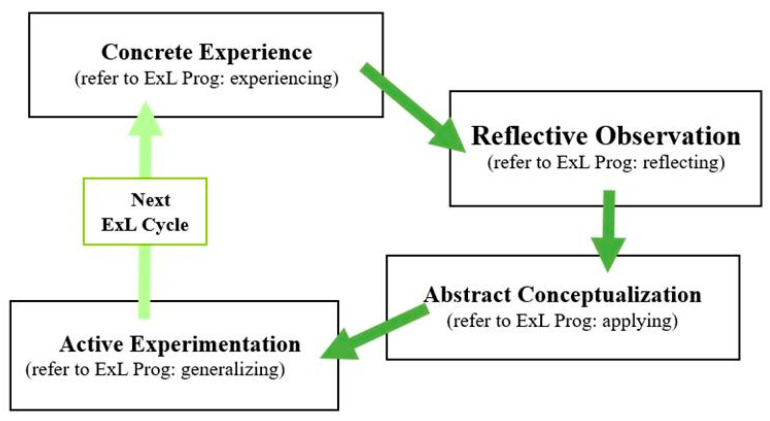
ExL Prog experiential learning cycle.

The core philosophy of ExL, ‘learning by doing’, posits that transforming life experiences through active engagement is key to successful learning [14,28,29]. The ExL theory introduces a cycle (the ExL cycle; Figure 1) that comprises four phases of experience and understanding, starting as individuals participate in activities, thus generating ‘concrete experiences (referred to as experiencing)’. They then observe and reflect on the activity process, a phase known as reflective observation (referred to as reflecting). Next, individuals deepen their understanding or reconstruct what they have learned, a phase known as abstract conceptualization (applying). Finally, they apply insights from these experiences to practical situations and adjust their behavior for greater adaptability in a phase known as active experimentation (referred to as generalizing; [11,13,14,15]). The ExL theory asserts that this cycle is a dynamic progression, where individuals continuously advance through these four phases in a spiral manner, thereby gaining a more comprehensive understanding of their experiences and enhancing their ability to apply what they have learned practically.

Some scholars suggest that applying the ExL framework to interpersonal interaction programs (referred to as the ExL Prog in this study) in inclusive settings can yield a wide range of profound collaborative experiences [4,5]. By reflecting on interpersonal feelings and behaviors and adopting new interaction patterns during the ExL cycle, individuals enhance their interpersonal skills [14,30]. Essentially, ExL Progs, through structured activities, enrich the self-awareness, empathy, and cooperation of individuals. Individuals can establish more adaptive and open-minded interpersonal interaction patterns.

### 1.3. The ExL Prog to Boost Peer Interaction and Acceptance

Relevant studies explore the ExL approach as a comprehensive framework for intergroup contact, aimed at comprehending the learning process and developing skills through reflection and action [14,15]. ExL Prog focus on understanding and applying the effects of interactions and collaborative activities [17,31], prioritizing reflection on real-life interpersonal interactions to enhance social skills through various hands-on experiences [15,29]. Furthermore, educational and developmental researchers apply the ExL Prog in inclusive settings to enhance educational outcomes. These intervention activities encompass peer tutoring and acceptance programs [32,33], social relationship maps and peer network interventions [17], and picture book teaching [34,35], each contributing to enhanced interpersonal interactions and acceptance. They discovered that implementing an ExL Prog through collaborative activities in inclusive settings benefits both students with special needs and their peers.

Accordingly, the ExL Prog promotes initial steps in peer interpersonal interactions in inclusive environments, demonstrating that such programs and related activities effectively initiate interactions between students without special needs and those with special needs. The ExL Prog also contributes to the subsequent co-occurrence of positive interaction and acceptance by gaining insight from reflection or applying these understandings to improve the quality of relationships between both groups [6,16,36]. Overall, the ExL Prog highlights how individuals can shape their perceptions of those with disabilities. Both parties learn from interacting with each other and become more understanding and accepting.

While the studies above demonstrate the effectiveness of the ExL Prog in inclusive settings for students with and without special needs, research on the application of the ExL Prog concerning peer interaction and acceptance is scarce, particularly among senior-grade elementary school students [17,31]. Considering that children actively reflect on interpersonal interactions, they often form a second- or third-person perspective [37,38]. This study investigated the effectiveness of the ExL Prog in promoting interaction relationships and acceptance attitudes in an inclusive environment for senior-grade elementary school students. The study anticipates that the ExL Prog will encourage understanding among students without special needs towards their peers with special needs and foster mutual assistance and acceptance during activities. It also expects the program to boost overall positive experiences of compassion and friendship relationships in both directions.

The study’s research questions were as follows: (1) What changes in ‘interaction relationships’ and ‘acceptance attitudes’ between students without special needs and students with special needs result from the ExL Prog (quantitative analysis)? (2) What are the experiences of students without special needs and students with special needs during the ExL Prog (qualitative analysis)? (3) After the ExL Prog, what are the experiences of students without special needs regarding ‘interaction relationships’ and ‘acceptance attitudes’ towards students with special needs (qualitative analysis)? (4) What are the feelings and thoughts of the classroom teachers of the participating students regarding the intervention’s effects after the ExL Prog (qualitative analysis)? The study investigated the practical effects of the ExL Prog on peer interaction and acceptance among students, as well as the evaluations of participating students and teachers regarding this intervention method.

## 2. Method

### 2.1. Study Design

To address the four research questions outlined above, we employed a mixed-methods approach that combined surveys and interviews with both quantitative and qualitative analyses. Quantitative data were derived from two questionnaires, and qualitative analysis was gleaned from feedback and interviews.

### 2.2. Procedures

We employed a quasi-experimental design without randomization research and divided students into two groups: an intervention group and a comparison group during the COVID-19 period. Each group comprised eight students without special needs and one student with special needs. The intervention group participated in the ExL Prog, whereas the comparison group continued with their regular activities. Quantitative data were collected through a questionnaire administered four weeks after the experiential activity intervention, and students’ responses were analyzed based on their scores. Qualitative data were collected through interviews conducted after the intervention. Furthermore, the qualitative data from the interviews were compared with the quantitative data gathered from the completed questionnaires.

### 2.3. Participants and the Setting

In total, 18 sixth-grade elementary school students aged 11–12 years in Taiwan were recruited, including 2 students with special needs (Table 1). The students with special needs (1) were enrolled in an inclusive classroom, (2) shared the same disability category (both had autism spectrum disorder), and (3) did not experience academic learning difficulties but had special needs in interpersonal interactions. The students without special needs (1) were in the same class as the students with special needs, (2) did not have academic learning difficulties, and (3) did not exhibit overtly friendly or unfriendly behavior in their interactions with the students with special needs. A teacher from the same school ensured the proper execution of the intervention activities within the ExL Prog. The researchers interviewed three classroom teachers to see if the students maintained the effects of the intervention after returning to their original classrooms. The first author, who has special education training, implemented the ExL Prog.

The schools participating in this study are located in suburban areas, with a total of 25 classes (including one resource class) for grades 1 to 6, and are considered small public schools. Among the participants, the students with special needs are all Taiwanese, with one indigenous student in both ExL Prog. and non-ExL Prog. groups. The students with special needs rank in the bottom 3% of academic performance, while their peers’ academic performance range between the top 3% and 10%. However, all participants speak Mandarin. The two students with disabilities in this study come from lower-middle socioeconomic backgrounds. In addition, there are four students from low socioeconomic backgrounds and four from lower-middle socioeconomic backgrounds in both ExL Prog. and non-ExL Prog. groups.

### 2.4. The ExL Prog

The ExL Prog was conducted over 4 days during the summer, consisting of two activities each day, culminating in a total of eight intervention activities (Table S1 presents Supplementary Information). The researchers divided all students into two groups: those participating in the ExL Prog, which involved intervention activities during a regular 40 min summer course, and those who did not receive any intervention, engaging solely in regular summer 40 min course activities.

The program is modelled after the ExL cycle, as proposed by Kolb [11,13], and further developed by Morris [14]. It guides students through a series of activities aimed at promoting interactions with peers. The process begins with interactive activities, which leads students to recall and reflect on these interactions and experiences, with the goal of distilling key insights and lessons learned. Subsequently, students engage in discussions to strategize adjustments and improvements for future interactions, with the overarching aim of fostering positive relationships and enhancing attitudes of acceptance.

A total of eight thematic activities within the program guided the students through four distinct phases of the ExL cycle. In Activity 1, students initially immersed themselves in the activity (Table S1). After concluding, children progressed to the second to fourth phases (experiencing, reflecting, applying, and generalizing), engaging in reflective review and extended thinking. They explored improvements in interpersonal skills and potential positive changes for the future, and sought sustainable methods for improvement.

To confirm the efficacy of the ExL Prog, the researchers recorded all eight activities. A teacher from the same school selected three of the eight activities to assess adherence to the 12 elements of the four phases within the ExL cycle. The percentage was calculated by dividing the number of items that matched the assessor’s selections by the total number of items, then multiplied the result by 100%. This calculation indicates a high level of compliance, with a degree of conformity of 92%.

### 2.5. Instruments

Interaction Relationship and Acceptance Attitude Questionnaires. After reviewing the literature on children’s interpersonal interactions and peer acceptance, the researchers developed a two-part questionnaire covering interaction relationships and acceptance attitudes (Table S2) that employs a four-point Likert-type scale, where higher cumulative scores indicate better interaction relationships or more favorable acceptance attitudes. The researchers asked five experts to review the content and wording of the draft to establish expert validity. The ‘Interaction Relationship Questionnaire’ had 22 items, and the ‘Acceptance Attitude Questionnaire’ had 20 items. After the content and wording were reviewed by five experts (with expertise in the well-being of individuals with disabilities, peer interaction and acceptance, and elementary education and counselling), the researchers eliminated inappropriate items. The final version of the ‘Interaction Relationship Questionnaire’ had 21 items, categorized into ‘Affective Experiences’ and ‘Interaction Performance’, comprising 7 and 14 items, respectively. The ‘Acceptance Attitude Questionnaire’ had 17 items, categorized into ‘Cognitive’, ‘Affective’, and ‘Behavioral’ aspects, comprising four, seven, and six items, respectively. Due to the limited reading comprehension ability of the student with special needs, the researchers administered the questionnaire only to the students without special needs after the completion of the ExL Prog.

Feedback Form and Interviews for The ExL Prog with Activities. To gather feedback from the students, one week after the completion of all eight activities of the ExL Prog, researchers conducted a semi-structured interview using the ‘Experiential Activity Feedback Form’ to gather reflections from all nine participants, including one with special needs, following each ExL activity. This feedback aims to capture the participants’ insights into the activities, providing information about what they have learned. It also serves as a basis for adjusting the content of subsequent activities. In addition, the researchers employed additional semi-structured interviews using the ‘Student and Teacher Interview Form’. The interviews focused on two aspects: interaction relationships (involving both students without special needs and students with special needs, totaling nine interviewees) and acceptance attitudes (specifically from students without special needs towards students with special needs, totaling eight interviewees). Moreover, interviews were conducted with the homeroom teacher and two subject teachers after the completion of the ExL Prog. The interviews aimed to compare the evaluations of interaction outcomes and peer acceptance between students and teachers, ensuring consistency. The first author (who was also a special education teacher at the school) conducted one-on-one interviews to ensure that their perspectives and ideas would not influence each other.

### 2.6. Data Collection and Analysis

Descriptive statistics were used to present the quantitative data from the Interaction Relationship and the Acceptance Attitude Questionnaires. Further statistical comparison of the differences in positive changes among the students without special needs with and without the ExL Prog was conducted. Qualitative data consist of feedback forms from all students who participated in the ExL Prog and interviews from the students without special needs who participated in the ExL Prog, as well as their homeroom teachers and two subject teachers. Qualitative data were employed to complement the limitations of quantitative findings. A thematic analysis approach [23] was utilized to identify recurring concepts among participants through in-depth examination of interview content, thereby enhancing the interpretation and presentation of results. After the first author completed the transcription, the interview content was validated with the participants to ensure accuracy. An initial analysis was then conducted, followed by a cross-verification of the qualitative data with the corresponding author to ensure the accuracy of the coding. Consensus on the results of the analysis was achieved, with an agreement rate exceeding 90%.The organized descriptive information is coded with T1 to T3, representing teacher codes; students without special needs are coded as S1 to S8, and the student with special needs is coded as S9.

### 2.7. Ethics Declarations

The study received approval from the university’s thesis review committee and was supported by a grant that had undergone review by the university’s Institutional Review Board. All participants provided written informed consent before participation. The authors declare no competing interests. The data that support the findings of this study are available from the corresponding author upon reasonable request.

## 3. Results

### 3.1. Group Comparison on Peer Interaction and Acceptance

To address the first research question, Table 2 and Table 3 present descriptive statistics from the Interaction Relationship and Acceptance Attitude Questionnaires. The total score of positive interactions in the intervention group, as well as the average values for the two dimensions (affective experience and interaction performance), were both lower than those of the control group. However, after analyzing the median, it was found that the total score of positive interactions and the scores for the two dimensions (affective experience and interaction performance) in the intervention group was higher than those of the control group. It is speculated that this discrepancy may be due to the small sample size of only eight students in both the intervention and control groups, where the influence of extreme values might be more pronounced.

**Figure 2:**
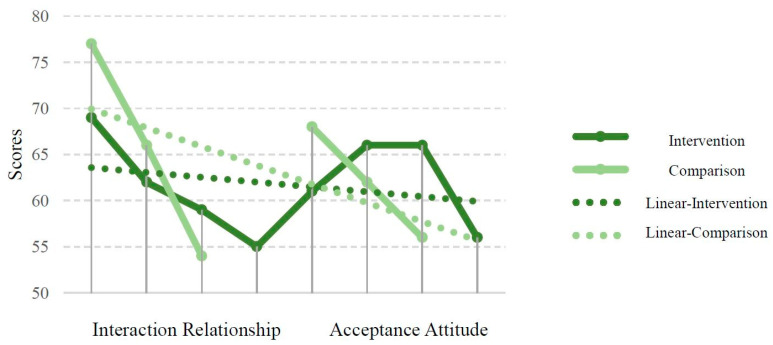
Trends in score distribution of above-average students.

The “cognitive” dimension of students without disabilities’ acceptance attitude toward students with special needs was lower than that of the control group, while the “affective” dimension was higher than that of the control group. The “behavioral” dimension was the same as that of the control group. Overall results indicated an equal distribution of high scorers between the two groups, suggesting no difference in population distribution. Notably, Figure 2 highlights a significant index that, compared with the comparison group (interaction relationships and acceptance attitudes: range = 23 and 12, *SD* = 11.50 and 6.00), the intervention group’s scores exhibit more central tendency (interaction relationships and acceptance attitudes: range = 14 and 10, *SD* = 5.91 and 4.79). This suggests that the ExL Prog may enable students without special needs to consistently improve in their interaction relationships and acceptance attitudes.

### 3.2. The Experiences of Cultivating Relationships and Acceptance During ExL Prog

Table S3 presents examples of the feedback from participating students who participated in the ExL Prog after each activity. The students’ feedback illustrates considerable development in experience, interactive relationships, and acceptance attitudes throughout the eight activity units. Students preliminarily encountered and reflected on their interpersonal reactions during the initial ExL Prog. They advanced towards more profound experiential reflections and the assimilation of diverse perspectives in the latter stages of ExL Prog. Quotes such as ‘Sometimes the advice we give may not be what the other person needs; we should try to understand their needs first and then offer advice to help them’ (S3) and ‘I learned to accept others and observe them with care’ (S2, S7, S9) highlight transformative experiences. This awareness suggests that students have shifted their viewpoints and undergone substantial transformations through repeated practices, leading to the refinement of their relationship concepts and increased consideration of others.

### 3.3. The Experiences of Navigating Positive Interaction and Promoting Peer Acceptance After the ExL Prog

Students without special needs participating in the ExL Prog reported positive experiences and enjoyed the activities. Many students gained insight into the significance of observing the requirements of students with special needs during these activities. In addition, they demonstrated compassion along with empathy towards the student with special needs by understanding his challenges from his perspective. For instance, one student shared, ‘When engaging with him [the student with special needs], I make a conscious effort not to overstep boundaries. I take into his limits and try hard to not annoying him intentionally’ (S5). Another student echoed, ‘When he [the student with special needs] refused to cooperate with the activity, I felt very angry… If a teacher reminds me to be mindful of his needs, I will speak to him appropriately, but I might still be accustomed to speaking loudly, as I did before’ (S6).

Previously, students without special needs who participated in the ExL Prog exhibited lower acceptance towards their classmates with special needs. However, their acceptance of these students noticeably transformed through participation in the ExL Prog. For example, one student remarked, ‘I would think he [the student with special needs] was intentionally causing trouble before, but now I try to think about why he would do that’ (S5). Another student also shared, ‘I might have thought that it’s his [the student with special needs] business in the past, but I understand that it’s just a matter of differences between us’ (S4). Another student commented, ‘Now, I’ll engage with him [the student with special needs] in a new way, aiming to discover and learn more about him [the student with special needs]’ (S7).

The student with special needs (S9) felt relaxed and less pressured in the group interactions. He learned the importance of valuing different perspectives, which led him to shift from previous reactions of anger and unwillingness to adopt the suggestions and opinions of classmates. The student (S9) shared: ‘When my peers are trying to be friend with me, I feel comfortable and enjoy being with them’ and ‘I learned to be more cooperative with others, and I realized that I should take others’ thoughts and feelings into considerations’.

### 3.4. Teacher Interviews on Positive Class Dynamics and Peer Support

The ExL Prog influenced class dynamics, enabling a shift from passive to proactive assistance among students without special needs towards their peers with special needs. A teacher noticed, ‘After the ExL Prog, the students without special needs began using a softer tone to address issues caused by the special needs student’ (T1). Another teacher commented, ‘In the class, conflicts involving the student with special needs were common prior to the intervention. Yet, I’ve observed increased maturity among the students without special needs. They demonstrated more initiative in assisting the student with special needs when they see him not following classroom rules or team rules’ (T2 and T3). Generally, disruptive relationships and rejection among students decreased, leading to greater patience and cooperation in peer interactions. Conflicts were replaced with mutual understanding and acceptance.

## 4. Discussion

The quantitative data show that the median scores of intervention group’s positive interaction scores between students with special needs and peers without special needs were higher than those of the control group. However, the intervention group’s acceptance scores were lower than those of the control group, meaning that the effect of the ExL Prog on improving peer acceptance was less significant, except for the “Affective” dimension, which was higher than that of the control group. Based on the results shown in Figure 2, the scores of the intervention group demonstrate a more central tendency than those of the control group, indicating reduced polarization and greater consistency in participants’ perceptions. This more balanced distribution may help explain the relatively lower acceptance scores, as increased exposure to the ExL Prog could have fostered more reflective attitudes among students. The qualitative data show that students without special needs in the intervention group reported a change in how they viewed peers with special needs through the ExL Prog. Their perspective shifted from negative tolerance to positive understanding, and they also learned to observe the needs of others before interacting. Students with special needs in the intervention group expressed that they learned to respect different opinions and were happy to have more opportunities to play with their classmates. The interviews predominantly reflected positive feelings and interaction styles.

This study found that the ExL Prog can enhance positive interaction between students without special needs and students with special needs. Throughout the ExL Prog, students without special needs reported their own attitudes toward their peers with special needs shifted from impatience to positive understanding and compassion. The students without special needs learned to observe each other’s needs and cooperate consciously. The student with special needs not only was pleased to have more opportunities to play with his peers through these constructive interactive experiences but also developed consideration for others’ perspectives. Regarding the effects on peer acceptance attitudes, the students without special needs indicated positive changes in their views toward the student with special needs after the ExL Prog. They learned to be respectful of the unique characteristics and difficulties for students with special needs in case of misunderstandings. In addition, both the homeroom teacher and subject teachers held positive views on the ExL Prog. Through intensive and comprehensive engagement in the ExL Prog, both students without special needs and students with special needs reacquainted themselves. Then, they became willing to listen, understand, and assist each other. This added value of change benefits teachers in class management by alleviating the burden of handling peer interaction and acceptance issues while enhancing cohesion among classmates.

The overall findings of this study demonstrate the effectiveness of the ExL Prog in enhancing positive interactions and acceptance between students without special needs and students with special needs. The program is consistent with the ExL theory [11,13,14] and previous related research [6,16,36]. The ExL Prog promotes structured participation in students across four distinct phases within the ExL cycle. Starting with immersion in the activity, students then transition to the subsequent phases, which involve reflective reviews, and engage in extended cognitive deliberation. This process enables the exploration of advancements in interpersonal aptitudes and the anticipation of prospective positive transformations. Ultimately, it creates a sustainable avenue for fostering a cohesive and inclusive atmosphere. More importantly, this study further emphasizes the critical role of senior-grade elementary school children in the bidirectional perspective of ExL [15,17,31], presenting more age-specific and comprehensive intervention effectiveness.

### 4.1. Practical Implications

#### 4.1.1. Advocating for Empathy and Respect Towards Differences

The sixth-grade students without special needs and students with special needs participating in this study’s ExL Prog fostered an appreciation for the uniqueness of each individual. The ExL Prog meaningfully transformed their peer interactions, enabling students to reconnect and reshape their interpersonal dynamics. Inclusive classrooms must promote students without special needs’ awareness and understanding of the challenges faced by students with special needs [4,5,6]. This enhanced awareness encourages students without special needs to develop an empathetic and respectful mindset that motivates them to provide support and compassion to those with visible physical or sensory impairments, as well as hidden cognitive or emotional disabilities. Likewise, students with special needs profit from increased peer resources and opportunities for positive outcomes. Hence, inclusive classrooms should prioritize refining a culture of understanding and acceptance through programs like ExL, rather than mere compliance.

#### 4.1.2. Enhancing Positive Dynamics in Inclusive Classrooms with the ExL Prog

Based on our findings and positive feedback across students with and without special needs, and their teachers, the ExL Prog could be used in inclusive education. The ExL Prog prompted the students to provide students with special needs with time to adapt, to kindly acknowledge differences, and to constructively balance mutual respect. A designed ExL Prog can promote peer interactions and acceptance among both students with special needs and students with special needs. By encouraging the recognition and understanding of differences, these activities lead to increased mutual assistance [4,5,39]. Additionally, using group cooperative work allows students to engage in experiencing, reflecting, applying, and generalizing [14,15]. To ensure effective program implementation, instructors should have a basic understanding of students with special needs and the dynamics between students without special needs and students with special needs.

### 4.2. Limitations and Future Research

The focus of this study on interpersonal interactions meant that participants’ interaction relationships could not be randomly assigned based on their inherent characteristics. As students with special needs experienced difficulties in comprehending the questionnaire items, their quantitative data were not included in the current study. This limitation should be addressed in future research. Moreover, the absence of a pretest for interaction relationships and the sole reliance on the statements of class teachers as references indicate that rigorous control of variables was not implemented. It is possible that the intervention group started with lower baseline acceptance levels, which could have influenced post-intervention outcomes. This suggests that researchers should interpret the research results with caution to account for potential interference or covariate factors. Since this research was conducted during the COVID-19 pandemic, it was not possible to carry out a pilot study in advance to assess the feasibility of the program. It is recommended that future studies include a pilot phase to better evaluate and refine the program design before full implementation. Additionally, the relatively small sample size and limited diversity in cultural backgrounds may have reduced statistical power, making it more difficult to detect subtle changes and potentially contributing to the central tendency observed in the score distribution. As a result, the interpretation and application of the research findings should be approached with caution, and further studies are needed to provide supporting evidence. Furthermore, the intervention timeframe of the ExL Prog was short, and the sustainability of its effectiveness still requires further confirmation in future research. Lastly, although our activity design in the program accounted for various aspects of ExL, it was limited to only eight activities, serving as instructional references. Future research could incorporate a variety of activities to apply the ExL Prog to enhance the interpersonal relationships and acceptance attitudes among students with and without special needs.

## 5. Conclusions

These findings are consistent with Kolb’s ExL theory [11,13,14], which emphasizes the importance of concrete experiences and reflective observation in shaping learning. The students involved in the ExL Prog recognize the importance of understanding others and have reflected on how their previous approaches might be ineffective in fostering relationships. Their consideration for others has been cultivated, but the transition from knowing to implementing these newfound skills sometimes demands ongoing support and reinforcement. These changes led to a newfound openness for interactions and goodwill from their peers, highlighting a growing sense of mutual reciprocity. Overall, the ExL Prog promoted transformative growth in interpersonal relationships and communication skills for the students. Future research should consider both sample representativeness and inferential validity to more thoroughly investigate the potential correlation between students’ positive interactions and their attitudes toward peer acceptance, as well as the extent to which these factors may mutually influence one another.

## Figures and Tables

**Table 1:** Comparison of Peer Interaction in Intervention and Comparison Groups in the Same Class.

Condition	Category	Peer Interaction
ExL Prog	ASD (boy, *n* = 1)	1. Often experiences conflicts and quarrels with classmates. 2. When reminded by classmates, the student often does not respond and continues behaviors that lead to further conflicts. After multiple conflicts, peer relationships deteriorate. 3. Is perceived by peers as intentionally uncooperative due to an invisible disability.
	General (boy, *n* = 5; girl, *n* = 3)	Exhibits subdued participation in class, rarely provides suggestions, and generally assumes a more passive role.
Non-ExL Prog	ASD (girl, *n* = 1)	1. Few conflicts with peers, though experiences a history of rejection and frustration in forming friendships. 2. Often engages in solitary activities, such as walking or reading after class. 3. Peer interactions mainly revolve around academic discussions, with less engagement in playful or casual interactions with this student, due to an invisible disability.
	General (boy, *n* = 4; girl, *n* = 4)	Actively participates in class, is proactive, willing to provide suggestions, and is considered a more active participant in the class.

**Table 2:** Descriptive statistics of general education students’ Interaction Relationship Questionnaire.

Statistics	Intervention (*n* = 8)			Comparison (*n* = 8)		
	Total	Affective Experiences	Interaction Performance	Total	Affective Experiences	Interaction Performance
Mean	51.75	15.50	36.25	53.75	16.75	37.00
SD	11.20	4.11	8.14	12.20	4.17	8.37
Median	51.00	16.50	37.50	50.50	15.50	34.00
Minimum	37.00	9.00	28.00	40.00	12.00	28.00
Maximum	69.00	22.00	47.00	77.00	25.00	52.00

**Table 3:** Descriptive statistics of general education students’ Acceptance Attitude Questionnaire.

Statistics	Intervention (*n* = 8)				Comparison (*n* = 8)			
	Total	Cognitive	Affective	Behavioral	Total	Cognitive	Affective	Behavioral
Mean	55.00	13.75	22.13	19.13	55.38	14.38	21.13	19.88
SD	8.73	1.17	4.32	3.72	7.09	1.85	3.64	2.75
Median	54.00	14.00	21.00	19.50	55.0	15.00	20.00	19.50
Minimum	44.00	12.00	17.00	14.00	45.00	11.00	18.00	16.00
Maximum	66.00	16.00	28.00	24.00	68.00	16.00	28.00	24.00

## Data Availability

The original contributions presented in the study are included in the article, further inquiries can be directed to the corresponding author.

## Supplementary Materials

The following supporting information can be downloaded at: https://www.mdpi.com/article/10.3390/children12050543/s1, Table S1: The ExL Prog Activities and Core Objectives; Table S2: The Interaction Relationship and The Acceptance Attitude Questionnaires; Table S3: Feedback Summary of General Education Students in the ExL Prog Intervention Group.
