# Supplementary material for: Promoting Peer Interaction and Acceptance Among Students with Special Needs Through an Experiential Learning Program
Source: Children (Basel). 2025 Apr 24;12(5):543. doi: 10.3390/children12050543 (PMC12109870; doi:10.3390/children12050543)
Supplement: Supplementary file 1 [file children-12-00543-s001.zip › children-3570316-supplementary.pdf]

Table S1

The ExL Prog Activities and Core Objectives

Theme	Activities *(time)*	Contents	Core Objectives
Theme 1: Recognize the difference	1. Discover your needs *(Day1, 10 mins)*	Using the tools available, help others pass items by observing and imagining how to collaborate using each person's tools to complete the item transfer.	Discovering each person's strengths and recognizing the needs of others
	2. See your world *(Day1, 10 mins)*	In pairs, one person without a blindfold leads and guides the other person with a blindfold to complete a task.	Understanding the difficulties of others and accepting differences
Theme 2: Empathy	3. Number queue *(Day2, 10 mins)*	In a scenario where individuals do not know their assigned numbers but can see everyone else's numbers, complete a queue in numerical order by observing each other's body language.	Cultivating empathy and understanding others' perspectives
	4. I protect you *(Day2, 12 mins)*	In a dodgeball game, the protector ensures that the person being protected is not hit by the person throwing the ball.	Cultivating empathy and understanding others' perspectives
	5. You tell me what to draw *(Day3, 15 mins)*	In pairs, one person acts as the drawer, and the other person can only verbally describe to guide the drawer in creating a target pattern.	Cultivating empathy and understanding others' perspectives
Theme 3: Recognize the difference and empathy	6. Hunter and prey *(Day3, 10 mins)*	Form a circle, and throw handkerchiefs towards the person standing in the middle. The person in the middle tries to catch as many handkerchiefs as possible.	Building empathy and recognizing difference
	7. Cooperative puzzles *(Day4, 15 mins)*	Multiple puzzle sets are divided into different envelopes. Without speaking, individuals actively offer puzzle pieces to others to achieve the final goal of each person completing a puzzle set.	Team collaboration and observing the needs of others
	8. The way forward	Team discussion on how to take turns challenging and navigating through the visible	Understanding tangible and

Theme	Activities *(time)*	Contents	Core Objectives
	(Day4, 20 mins)	and hidden obstacles in a maze, completing the maze game in the end.	intangible barriers and empathizing with difficulties.

Table S2

The Interaction Relationship and The Acceptance Attitude Questionnaires

**Interaction Relationship**	**Question number**	**Content**
emotional feelings	1	When helping him express his ideas, I will listen carefully.
	2	I enjoy sharing stationery with him.
	3	I like it when he helps me.
	4	I feel relaxed and happy when chatting with him.
	5	I would be very happy to do the same things as him.
	6	I don't like watching him perform on stage.
	7	I dislike him touching my things. (reversed statement)
interaction performance	8	During group activities, I will take the initiative to be in the same group as him.
	9	I will lend him my toys.
	10	When working together, I can involve him in completing certain parts.
	11	If needed, I will help him.
	12	When he talks to me, I will listen carefully.
	13	In games, I can treat him fairly.
	14	If given the chance, I'm willing to attend the same class as him.
	15	When he is sad, I will ask him what's wrong.
	16	When he faces difficulties, I will help him.
	17	I will share with him about what's happening in my life.
	18	I will gently remind him of things he hasn't completed yet.
	19	I will share topics of mutual interest with him.
	20	Unless the teacher asks for my help, I won't proactively assist him. (reversed statement)
	21	If I encounter him at school, I will pretend not to see him. (reversed statement)
**Acceptance Attitude**	**Question Number**	**Content**
cognitive	1	I feel that everyone needs help at times.
	2	I believe spending time with him has taught me to see things from another perspective.
	3	I think each classmate is unique.
	4	When he faces setbacks, I can understand his feelings.
affective	5	I would gladly assist him.
	6	If I'm wrong, I'm willing to apologize to him.
	7	I'm willing to listen to his feelings.
	8	I'm willing to be in the same group as him.
	9	I'm willing to proactively show concern for him.
	10	I dislike sitting next to him.
	11	I dislike studying in the same class as him. (reversed statement)
behavioral	12	I would tell my good friends to respect him.
	13	I won't play pranks on him.
	14	When he doesn't understand what I'm saying, I will try to explain it in a

	way he can comprehend.
15	When he speaks on stage, I will listen attentively.
16	If he helps me, I will express my gratitude proactively.
17	I dislike reminding him of things he should do. (reversed statement)

Table S3

Feedback Summary of General Education Students in the ExL Prog Intervention Group

Activities	Feedback
1. Discover your needs	Feeling good when being helped. (S4、S2)
	Finding some tasks challenging. (S3、S6、S7、S9)
2. See your world	Feeling nervous as my classmates in the same group couldn't understand my directions. (S1)
	Classmates hesitated to move forward because they couldn't see, making it challenging for me to assist. (S2、S4)
	Assisting in guiding others was difficult; sometimes, I also got confused. (S3、S5)
3. Number queue	Finding the activity a bit challenging but enjoyable. (S2、S5、S4、S7、S3)
	Actively listening and understanding others' commands are crucial. (S6)
	I helped others organize the order first, then listened to suggestions from others. (S1)
4. I protect you	Believing that anyone with the ability can protect others. (S3)
	Although not skilled at protecting others, I would still do it when needed. (S1)
	Finding the game thrilling, tense, and enjoyable. (S2、S4、S5、S6、S7、S8、S9)
5. You tell me what to draw	I find that sometimes I am quite firm in sticking to my own answers. (S3)
	Both the person explaining and the person drawing may misunderstand each other's intentions. (S1)
	I notice that sometimes classmates misinterpret what I say, and I have to explain it again. (S4)
6. Hunter and prey	When the other person accepts my suggestions, I am more inclined to accept their suggestions. (S8、S6)
	When too many suggestions appear at once, I feel unsure about what to do. (S1、S7、S2)
	Playing the role of giving advice is a bit challenging because sometimes it's hard to find the right time to give advice—sometimes too fast, sometimes too slow. (S3、S5)
7. Cooperative puzzles	Sometimes the advice we give may not be exactly what the other person needs; we should try to understand their needs first and then offer helpful advice. (S3)
	From the activity, I learned to observe before helping others. (S1、S4、S5)
	When I see someone in need, I want to help them quickly. (S6、S8)
8. The way forward	Working together to help me find the right path made me feel lucky. (S4)
	Being unable to see the obstacles ahead made it difficult, and I needed the person in front to help me navigate obstacles. (S1)
	When no one helps, I have to guess, making it feel challenging. (S7、S8)
	I'm very happy that everyone worked together to find the right path forward. (S9)
